# Comparison of Surgical Blade and Cryosurgery with Liquid Nitrogen Techniques in Treatment of Physiologic Gingival Pigmentation: Short Term Results

**Published:** 2014-12

**Authors:** Saeed Rahmati, Mansoore Darijani, Maryam Nourelahi

**Affiliations:** a Periodontist, Isfahan, Iran.; b Postgraduate Student, School of Dentistry, Mashhad University of Medical Sciences and Health Services, Mashhad, Iran.; c Postgraduate Student, Department of Periodontology, School of Dentistry, Shahid Sadooghi Yazd University of Medical Sciences and Health Services, Yazd, Iran.

**Keywords:** Pigmentation, Physiologic, Cryotherapy, Surgical blade

## Abstract

**Statement of the Problem:** Melanin pigmentation of the gingiva is a crucial esthetic problem. A variety of methods have been used for gingival depigmentation.

**Purpose:** The purpose of this study was to compare the results of two treatment modalities: scalpel technique and cryotherapy with liquid nitrogen in treatment of gingival pigmentation.

**Materials and Method:** Twenty patients with chief complaint of gingival pigmentation participated in our study. 10 patients were treated with cryotherapy and remaining 10 participants were undergone the scalpel technique surgery. We evaluated acquiescence and comfort of the patients, degree of depigmentation, based on the area of pigmentation shown by gridlines option in Microsoft Paint software, and the presence or absence of gingival recession before and one month after treatment. Data was analyzed using Mann-Whitney and Chi-Square tests. A significance level of p≤ 0.05 was adopted.

**Results:** Mean value and standard deviation of depigmentation for group A and group B was 96.17±2.51 and 95±2.48, respectively. The difference was not statistically significant (p= 0.225). There was no association between the treatment modality and the gingival recession (p= 0.303) or the treatment modality and the patient satisfaction (p= 0.346). No significant difference was found between gingival recession measures before and after the operation in the two treatment modalities.

**Conclusion:** Surgical blade and cryosurgery with liquid nitrogen had no significant difference in treatment of physiologic gingival pigmentation. Both Techniques are acceptable in the treatment of gingival pigmentation.

## Introduction

Attractive smile is a crucial concern of today’s dentistry and the color of gingiva has an important impact on this issue. [1] Pigmentation is a change in the color of the gingiva and oral mucosa [2] and is considered as a foremost esthetic problem. Gingival pigmentation has two types: physiologic and non-physiologic (pathologic). [3] Melanin, a brown pigment, is the natural pigment causing the endogenous pigmentation of the gingival. [4]

Pigmentation usually appears in the gingiva in the first three years of life as a diffuse bluish discoloration or unlikely as the brown or light brown patches. [5] Various intensity and distribution of pigmentation is seen in the oral mucosa whilst the gingiva is the most predominant site. [2] Different factors affect the color of the gingiva such as the size and the number of blood vessels, epithelial thickness, rate of keratinization and the presence of epithelial pigments. [6] The normal color of the gingiva is coral pink, with a range of pale pink to bluish purple . The studies show that the most acceptable color of the gingiva among people is pale pink. [3]

The prevalence of pigmentation is more in labial gingiva than buccal, palatal and lingual gingival, [2] and is more frequently observed in the anterior region. [6] Physiologic gingival hyper pigmentation is seen as a genetic trait in some population regardless of the age and gender. [4] It can occur in all racial groups, but is more common in people with dark skin. [7]

Whilst gingival pigmentation is most commonly benign and features no underlying medical problem, it is considered as an esthetic problem particularly in people with high lip line. [8-9] Gingival pigmentation represents melanocytic hyperactivity, which usually occurs in attached gingival. [8-10] Melanin which is accumulated in the cytoplasm of melanocytes excretes and precipitates in the pigmentation process. [6, 11, 2] The degree of pigmentation depends on mechanical, chemical or/and natural stimuli. [11] Diffuse and multiple pigmentations are attributable to genetics, drugs, inflammation, and endocrine disorders. [2]

Gingival depigmentation is a periodontal plastic surgery in which the gingival hyper pigmentation is removed or reduced by various methods [4] such as: gingivectomy, electro surgery, cryosurgery, bur abrasion, Nd-Yag laser, and scalpel blade technique. [1, 12] Selecting any of these techniques depends on clinical expertise and personal preference of the clinicians. [13]

Scalpel blade technique is the most common method for the treatment of gingival pigmentation. [1] Gingival epithelium is scraped with a surgical blade or diamond bur and the remained connective tissue is healed by secondary intention. The regenerated epithelium would have no melanin hyperactivity. [14] No pain, scar or infection has been reported with this procedure. [13] The disadvantage of this technique is  bleeding during or after the procedure; hence, the surgical region requires to be covered up by a periodontal dressing for 7 to 10 days. [11] In thin biotype of the gingiva, cares should be taken not to expose the alveolar bone. This method is simple and cost effective; the esthetic results are usually acceptable and are recommended in developing countries. [15]

In cryosurgery, the gingiva is freezed with different materials such as liquid nitrogen. [8] This technique, which is considered as one of the most effective treatments for benign lesions, [16] is based on rapid freezing of water and slow melting repeatedly, leading to tissue deterioration. [17] The cryotherapy has some direct effects including crystal formation in intra and extracellular fluid, cell dehydration, enzyme inhibition, protein denaturation, and cell death due to thermal shock. It has also some indirect effects such as changes in vasculature and immune response of the tissue, which leads to cell death. [18]

Regarding the advantages of this method, this technique is easy and rapid to apply, does not require anesthesia or suturing, and finally it does not cause any bleeding or scars. [8, 19] The disadvantages of cryosurgery include soft tissue swelling, technique sensitivity and being harmful in casual touch. [19] The depth control is also difficult and the optimal duration of freezing is not known, and subsequently, prolonged freezing increases the tissue destruction. [13]

The aim of this study was to compare the results of cryosurgery and scalpel technique in treatment of physiologic gingival pigmentation. 

## Materials and Method

In this clinical trial, approved by ethics committee of Zahedan University of Medical Sciences, 20 patients (5 male, 15 female) with the age range of 10 to 31 years (mean 22.6 years) were included in this study. The patients were referred to Periodontics Department of Zahedan Faculty of Dentistry with chief complaint of gingival pigmentation. No systemic or environmental factors causing a pigmentation to occur such as endocrinopathy, smoking or taking drugs capable of inducing pigmentation were present in participants. Initial examination and a review of medical history confirmed physiologic pigmentation of the gingiva with suitable biotype, satisfactory oral hygiene, and plaque control. Exclusion criteria of the study included: pigmentation induced by systemic or environmental factors, thin biotype of the gingiva, and good plaque control. Patients were given oral hygiene instructions and then were randomly divided into two groups: group undergoing “cryosurgery with liquid nitrogen” (group A) and the group enduring “scalpel technique” (group B).

**Cryosurgery procedure**

In this technique, the labial surface of the anterior segment of maxilla was isolated with cotton rolls and suction. Topical anesthesia with Lidocaine 10% was applied for 10 minutes. Roughly 20 mL of liquid nitrogen (Oxygen gas Khorakian Co.; Tehran, Iran) was used. The liquid nitrogen was carried to the department of periodontics in the morning of each surgery day. Liquid nitrogen was applied with a rolling motion for 20 seconds, using a liquid nitrogen-cooled cotton swab for 5 seconds. The temperature of liquid nitrogen was- 186^o^C. During the procedure, the patients were wearing protective glasses and the vital teeth were protected by a periodontal dressing.

**Scalpel blade technique**

**Figure 1:**
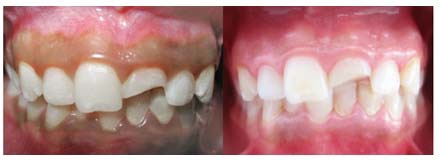
Before and one month after cryotherapy

**Figure 2:**
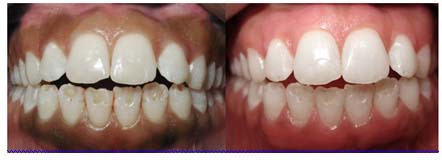
Before and one month after surgical blade technique

**Figure 3:**
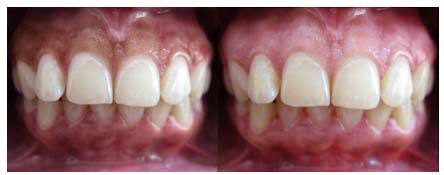
Before and one month after cryotherapy

In this method, labial surface of the anterior segment of the upper jaw was anesthetized with infiltration injection using 2% Lidocaine. A no.15 surgical blade was used to scrape the epithelium beneath the pigmented layer so that the visible pigmentation was entirely removed from margin of gingiva to the mucogingival junction. The bleeding was controlled by applying cold pressure with moist gauze. The exposed area was covered with periodontal dressing for 1 week. After the procedure, in both techniques, chlorhexidine 0.2% was prescribed twice a day for one week. During cryotherapy, care was taken to hold off the vital teeth from any contact with liquid nitrogen. To record and compare the data yielded from each individual, we took standard photographs the day before, and one month after the procedures. A digital camera (Canon, Japan) with 10 MP resolutions, ×4 magnification, flash off and macro lens was employed. The position of the patients, the distance to the camera, and the environmental lighting was similar for all patients before and one month after the procedure. [8] Two calibrated and blinded observers examined and compared the area of pigmentation in photographs before and after the procedures separately, using Microsoft Paint software version 6.1 in a LG F 700 Flatiron monitor (assembled in Iran). The area of pigmentation was determined using the gridlines, available in this software. Examples of the pictures taken before and one month after the operation are illustrated in Figure 1, 2 and 3.

Two calibrated and blinded examiners assessed the patients clinically before and one month after the procedures and recorded their opinion about the percentage of change in the area of pigmentation during this time. All the patients were examined with a periodontal probe to determine the distance between gingival margin and CEJ of the involved teeth before and one month after the procedure in order to detect any gingival recession relative to CEJ line. Patients were asked to fill out a questionnaire to evaluate their acquiescence and comfort right after the operation ended. They were asked to choose between excellent, very good, good, moderate, bad, and very bad regarding their compliance and comfort during the procedure. Statistical analysis was per formed by adopting SPSS-15 software. Mann-Whitney test was enrolled to analyze quantitative data and Chi-square test was used to analyze qualitative data. A significance level of p≤0.05 was considered. 

## Results

The results were evaluated by four observers, blinded to the type of the treatment. Two observers scrutinized the patient’s photographs and the other two examined the patients clinically. The results of treatment modalities on the area of pigmentation were summarized in Table 1.

**Table 1:** Mean value and standard deviation of depigmentation effects evaluated by 4 observers

**Treatment modality** ** ** **Observer**	**Cryotherapy (A)**		**Scalpel technique (B)**		**Mann Whitney** **test**
	**Mean ** **value**	**Standard ** **deviation**	**Mean ** **value**	**Standard ** **deviation**	
1 (clinical evaluation)	96.4	±3.13	94.5	±2.84	p= 0.035*
2 (clinical evaluation)	95.8	±2.49	95.9	±2.60	p= 0.373
3 (evaluation of the photographs)	96.3	±2.36	95.4	±2.17	p= 0.301
4 (evaluation of the photographs)	96.2	±2.53	95.1	±2.81	p= 0.377

* Statistically significant

In general, mean value and standard deviation for cryotherapy with liquid nitrogen and scalpel technique were 96.17±2.51 and 95.2±2.48, respectively. The difference was not statistically significant (p= 0.225).

The results also showed that four patients in group A (40%) and one patient in group B (10%) developed gingival recession. Based on the results of chi-square test, there was no relationship between the treatment modality and the gingival recession (p= 0.303). There was no relationship between the gingival recession before and after the operation in both treatment modalities. There was no relationship between treatment modalities and patients’ acquiescence and comfort. None of the patients expressed their opinion as moderate, bad or very bad. Data are summarized in Table 2.

**Table 2:** Patients’ acquiescence and comfort during the operation in two treatment modalities

**Patients ** **comfort** ** ** **Treatment ** **modality**	**Excellent**		**Very good**		**Good**		**Total**		**Mann-** **Whitney** **test**
	**Frequency**	**Percentage**	**Frequency**	**Percentage**	**Frequency**	**Percentage**	**Frequency**	**Percentage**	
Cryotherapy (A)	4	40	1	10	5	50	10	100	p= 0.246
Scalpel technique (B)	6	60	2	20	2	20	10	100	

## Discussion

This clinical trial was aimed to compare two treatment modalities for gingival depigmentation including scalpel technique and cryotherapy. The results of these treatment modalities were not statistically significant based on the findings yielded in this study.

The results of the current study were similar to the results obtained by the study of Khalid et al. that used scalpel technique for gingival. Their study included good patient satisfaction, excellent treatment results, and absence of postsurgical complications such as infection or scar. [15] Concurrent with our study, Kanakamedala et al. also treated gingival pigmentation by means of scalpel technique. Similar results included no postsurgical pain for any of the participants, no bleeding, infection or scar, acceptable healing, good patients’ satisfaction, and excellent treatment outcomes. [13]

In line with our study, Humangain et al., employed scalpel technique and has reported no postsurgical difficulties or complications, pink healthy gingiva with a normal consistency and patients' satisfaction. [14] Prasad et al. also achieved similar results such as satisfactory healing, no postsurgical pain or sensitivity and successful depigmentation with using scalpel technique for gingival depigmentation. [11]

Concerning the cryosurgery, our results were similar to the results obtained by Shirazi et al. in gingival depigmentation. The results included satisfactory treatment outcomes, absence of scar, infection, bleeding or defect after treatment and regaining normal gingival color in two weeks after the procedure. But unlike Shirazi et al. that reported no pain after the procedure, there were some pain reports by the patients of our study in 24 to 72 hours after the operation. [8] Similar results to our study were reported in the study of Shahbaz and Darbandi who used cryotherapy for the treatment of gingival pigmentation. [7] They found cryotherapy as a safe and an easy method, which caused no bleeding, infection, or scar. They also concluded that oral cavity was an appropriate environment for cryotherapy owing to the presence of saliva. [7]

The results of our study were in accordance with Tal et al.’s study that used cryotherapy for gingival  depigmentation. They reported this modality as an appropriate method and the normal gingival color was regained after 4 weeks. [9] MoeenTaghavi and Talebi used cryotherapy for treatment of gingival pigmentation.

They reported cryotherapy as a safe and acceptable method for gingival depigmentation which was similar to our results. [20] In another study, enrolled by Yeh CJ, cryotherapy was shown to be a simple method with no bleeding, which was comparable to our results. [21]

Bur abrasion and cryosurgery were compared in a study by Kumar et al. in which it was observed that healing after both techniques was uneventful and no scar was produced. [22] Mild degree of re-pigmentation was perceived in both techniques without any cosmetic significance on the 30th day of the observation, being in accordance with the results yielded by the present study. They found cryosurgical technique to be more superiorly acceptable for the operators and the patients, whilst we experienced both techniques being equally acceptable. [22] We applied liquid nitrogen for 20 seconds in each region since the available literature reported that the application of liquid nitrogen for 20-30 seconds achieved excellent outcomes. [7-8, 21]

In this study, the patients were asked about their symptoms during and after the operation. The patients who have undergone scalpel technique reported no symptoms during or after the operation and were absolutely satisfied. However, most of the patients reported a sensation of burning in their gingiva during and after 24-72 hours of cryotherapy, despite being known as a relatively painless procedure. This was probably due to the immediate blockage of the neural transmission in the area. [18]

In a case study reported by Özcan et al., various complications in cryosurgical procedures were reported such as infection, hemorrhage, recession and pain. [23] Pain was also reported by some of the patients in our study but the other complications were not conveyed. Previous studies [12, 24-25] have shown that the recurrence occurred with a high frequency and predominantly in a late stage; hence, long term studies are required for better evaluation of the treatment outcomes.

One of the limitations of this study was using cotton swabs for cryotherapy. The disadvantage was the absence of control over the temperature achieved within cells and the area of freezing that makes it hazardous to be employed intraorally, though being more available for clinicians. [ 26 ] Another limitation was that the two blinded observers, who were supposed to express their opinion about the degree of depigmentation, visited the patients clinically before and after the operation with an interval of one month which could have given rise to some inaccuracy in the study. Having two other observers analyzing the area of pigmentation in Microsoft Paint using the gridlines option would probably compensate for this predicament.

Good plaque control was defined as inclusion criteria in our study since less bleeding in a healthy gingiva and the easier tissue handling would result in more predictable treatment response. [27] Liquid nitrogen is relatively cheap and the other equipments requisite for cryotherapy is uncomplicated. This method is also easier and less technique sensitive than surgical approaches. It can be regarded as a less invasive method because there is almost no concern about losing keratinized tissue in this procedure. [8] We concluded that both treatment modalities were successful in gingival depigmentation; however the decision on selecting any of these two techniques is based on the clinician preference and expertise.

## Conclusion

Cryotherapy with liquid nitrogen and the scalpel technique were both effective methods for treatment of gingival pigmentation. Comparing these two treatment modalities, the treatment outcomes such as patients’ comfort and acquiescence, resultant color of the gingiva, and gingival recession in one month after the procedure were not significantly different. There was no relationship between the treatment modality and the gingival recession and also between the treatment modality and the patients’ satisfaction. 
